# Effects of Long-Term Chatbot System Use on Healthcare Professionals' Professional Identity Formation and Stress: A Small-Scale Comparative Study

**DOI:** 10.7759/cureus.80373

**Published:** 2025-03-10

**Authors:** Yuusuke Harada

**Affiliations:** 1 Graduate School of Humanities and Social Sciences, Hiroshima University, Hiroshima, JPN; 2 Graduate School of Medicine, Chiba University, Chiba, JPN

**Keywords:** digital mental health, identity, job stress, mental health apps, mental issues in healthcare professionals

## Abstract

Background

Digital mental health interventions, including chatbot systems, are increasingly recognized for their potential to address mental health challenges among healthcare professionals. In particular, reflective practices facilitated by chatbots may support identity development and alleviate stress. However, the long-term effects of such interventions remain underexplored.

Objective

This study investigated the effects of a chatbot system using the line chart method over approximately nine months on the professional identity development and stress levels of healthcare professionals in Japan.

Methods

Professional identity formation specifically refers to how healthcare professionals perceive, develop, and integrate their professional roles and responsibilities into their self-concept. To evaluate this construct and associated stress levels, a parallel-group design was employed, in which eight participants (nurses and physical therapists) were randomly allocated to either a system-use group (Group A) or a non-use group (Group B). Both groups were followed for nine months, with periodic assessments conducted before and after the intervention, as well as after a washout period. The Japanese version of the Dimensions of Identity Development Scale (DIDS-J), assessing Commitment Formation, Identification with Commitment, Broad Exploration, Deep Exploration, and Ruminative Exploration, and the Public Health Research Foundation Stress Checklist Short Form (PHRF-SCL), evaluating Anxiety/Uncertainty, Fatigue/Physical Responses, Autonomic Symptoms, and Depressive Mood/Inadequacy, were administered.

Results

In the between-group comparisons, Group A demonstrated statistically significant improvements compared to Group B in the DIDS-J subscales, including Commitment Formation (16.5±0.6 vs. 14.0±0.8), Identification with Commitment (16.5±0.6 vs. 14.3±1.0), Broad Exploration (18.0±0.8 vs. 15.0±0.8), and Deep Exploration (18.0±1.1 vs. 14.5±1.3). Additionally, significant improvements were observed in the PHRF-SCL subscales, specifically Anxiety/Uncertainty (5.5±1.3 vs. 7.5±0.6), Fatigue/Physical Responses (4.5±0.6 vs. 7.8±1.3), and Depressive Mood/Inadequacy (4.5±1.3 vs. 9.3±0.6).

Conclusion

The results suggest that long-term use of a chatbot system employing reflective methods may promote professional identity development and reduce certain stress responses in healthcare professionals. Nonetheless, sample size limitations, pre-existing group differences, and environmental variables constrain the interpretation of findings. Future research with larger and more diverse populations, extended follow-up periods, and additional physiological or life-event measures is warranted to validate and refine these preliminary outcomes.

## Introduction

Digital mental health solutions are increasingly recognized as indispensable for addressing mental health challenges in Japanese society [1]. The integration of digital tools, including mobile health (mHealth) applications, telepsychiatry, and digital phenotyping, holds promise for bridging the gap between the demand for mental health services and their availability [2]. In Japan, mental health conditions such as anxiety and depression are particularly prevalent among the working population. According to data published by the Ministry of Health, Labour, and Welfare in 2011, 90.8% of large corporations reported having employees who took continuous leave for one month or longer due to mental health issues within the past year. Furthermore, 75.4% of companies reported employees resigning due to mental health concerns [3]. Therefore, digital interventions in the workplace are expected to provide scalable and accessible means of mental health support [4].

Among various mHealth applications, those incorporating cognitive behavioral therapy (CBT) have shown potential for alleviating anxiety and depressive symptoms among Japanese employees [1]. For instance, a randomized controlled trial evaluating the "INTELLECT" application demonstrated significant reductions in depressive symptoms and improvements in cognitive functioning among users compared to a control group [5]. Such digital platforms also facilitate the remote delivery of mental health services, offering highly personalized interventions that help address the stigma often associated with traditional mental healthcare [1], particularly in Japan, where stigma continues to restrict access to conventional services [6,7].

In this context, Harada et al. reported that reflective activities facilitated by chatbot systems might contribute to stress reduction and promote identity development: two factors demonstrated to be interrelated [8]. However, these findings are based on cross-sectional studies utilizing relatively brief interventions, leaving the long-term sustainability of these effects and subsequent psychological changes insufficiently explored.

Moreover, significant challenges remain to be addressed, such as ensuring user engagement with digital interventions [9,10], resolving ethical issues related to data privacy and security [11,12], and establishing more robust, evidence-based approaches for practical implementation [9]. Against this backdrop, the present study conducted a nine-month follow-up investigation using a chatbot system similar to that described by Harada et al., examining how long-term use impacts professional identity formation and stress reduction among healthcare professionals. Specifically, we aimed to clarify whether continued use of the chatbot system would lead to measurable changes in the subscales of the Japanese version of the Dimensions of Identity Development Scale (DIDS-J) and the Public Health Research Foundation Stress Checklist Short Form (PHRF-SCL), thereby elucidating the effects on professional identity and stress levels. Our findings are anticipated to provide important insights for the design of future digital mental health interventions.

## Materials and methods

Definitions

In this study, the term "identity" refers to a broad conceptualization that encompasses a sense of identity and is treated in agreement with "multidimensional identity." The concept of "professional identity formation" refers specifically to the professional domain in traditional identity formation and refers to how health professionals perceive, develop, and integrate their professional roles and responsibilities into their self-concept [13]. Commitment, which is considered a component of multidimensional identity, refers to aspects measured by the Japanese version of the DIDS-J [14]. The terms "pre- and post-intervention assessments" and "pre- and post-follow-up assessments" are used synonymously in this study.

Participants

Participants were healthcare professionals recruited on a voluntary basis from facilities in Hokkaido, Japan. Participation criteria included being a nurse, physical therapist, or other professional and willingness to use the chatbot system. Exclusion criteria included unwillingness to provide consent or inability to maintain participation for nine months. This study was conducted at Kosetsu Hospital in Hokkaido, Japan, after being reviewed and approved by the Chiba University Graduate School of Medicine Ethical Review Committee (approval number: M2012). Written informed consent was obtained from all participants.

Methods

**Figure 1:**
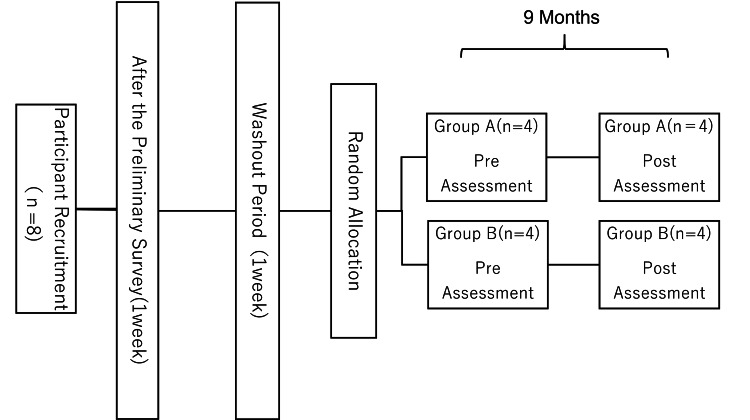
Study flow diagram

A parallel-group design was employed. The eight participants were randomly assigned to either Group A (system-use group) or Group B (non-use group). A one-week preliminary period was established to allow Group A participants to become familiar with the chatbot system, followed by a one-week washout period. The main intervention period extended from August 24, 2023, to May 7, 2024 (approximately nine months). Participants in the system-use group were instructed to use the chatbot system at least once per day for at least five days per week, while the non-use group was asked to continue their usual daily routines (Figure 1). The frequency of system use was verified by referring to system access logs.

**Figure 2:**
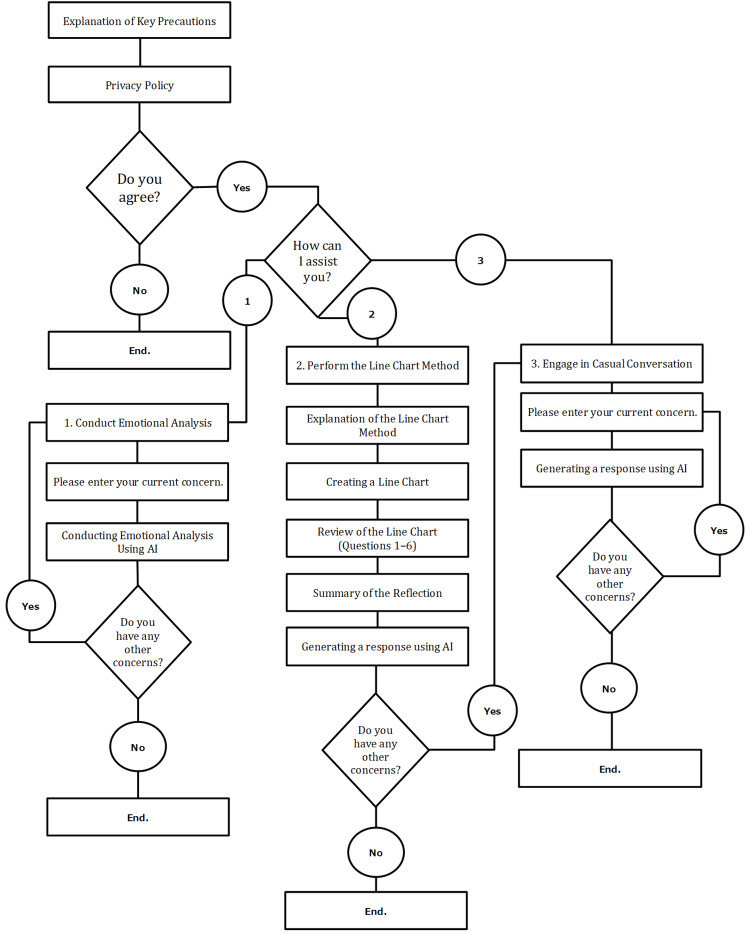
Overview of the chatbot system

The chatbot system utilized in this study offers three functions: (1) an emotional self-analysis feature, (2) a reflective tool based on the line chart method, and (3) an artificial intelligence (AI)-driven consultation tool for concerns. For the purposes of this research, only the second function, the line chart-based reflection tool, was employed (Figure 2). In the line chart method, participants reflected on their daily emotions and work experiences using visual charts. Participants rated their emotional state numerically and charted their daily reflections in order to visually analyze patterns and emotional trends over time.

DIDS-J [14] and PHRF-SCL [15] were administered in Japanese translations, recording and comparing identity and stress changes via a web-based platform before and after the intervention. Basic demographic information such as gender, age, and occupation were also collected.

For data analysis, a simple tabulation was performed on the demographic variables. To assess intervention effects, Shapiro-Wilk tests were used to verify the normality of each subscale of the two instruments post-intervention, followed by the independent samples t-test. Additionally, to track score changes from pre- to post-intervention, paired t-tests were conducted.

The DIDS-J scoring employed a 5-point Likert scale from 1 ("not applicable") to 5 ("strongly applicable") for each of the five subcategories, which were then tabulated. Note that higher scores indicate higher involvement in each item. Similarly, the PHRF-SCL for multidimensional identity formation uses a 3-point Likert scale ranging from 0 ("not applicable") to 2 ("strongly applicable") for each of the four subcategories, and both scales were treated as Likert scales in the analysis. All statistical analyses were performed using JASP software (Version 0.17.2, JASP Team, 2023), with a significance level of 5%.

## Results

The basic demographic characteristics of the participants are presented in Table 1. The mean(±SD) ages of Group A (system-use group) and Group B (non-use group) were 30.1±2.2 and 30.6±3.6 years, respectively. In Group A, three participants were male and one was female; in Group B, two were male and two were female. Participants in both groups were nurses or physical therapists.

**Table 1:** Basic demographic information and baseline scores for both groups *: p<0.05, independent samples t-test Mean±standard deviation DIDS-J: Dimensions of Identity Development Scale Japanese version; PHRF-SCL: Public Health Research Foundation Stress Checklist Short Form

Attributes and scales	Values (n=8)	
	Group A (n=4)	Group B (n=4)
Age (years)	30.1±2.2	30.6±3.6
Sex (male-to-female)	3:1	2:2
Occupation		
Nurse(s)	2	3
Physical therapist(s)	2	1
DIDS-J		
Commitment Formation	13.1±1.1	13.7±1.6
Identification with Commitment	12.4±0.8	12.7±1.1
Broad Exploration	16.1±2.1	15.6±1.6
Deep Exploration	16.2±1.2	15.4±0.8
Ruminative Exploration	17.8±2.1	18.3±1.4
PHRF-SCL		
Anxiety/Uncertainty	8.4±1.3	8.0±1.4
Fatigue/Physical Responses	7.8±0.8	7.6±2.1
Autonomic Symptoms	5.4±1.4	7.3±1.6*
Depressive Mood/Inadequacy	5.2±2.3	5.4±1.4

Regarding the DIDS-J subscale scores, Group A versus Group B comparisons yielded the following means(±SD): Commitment Formation: 13.1±1.1 vs. 13.7±1.6, Identification with Commitment: 12.4±0.8 vs. 12.7±1.1, Broad Exploration: 16.1±2.1 vs. 15.6±1.6, Deep Exploration: 16.2±1.2 vs. 15.4±0.8, and Ruminative Exploration: 17.8±2.1 vs. 18.3±1.4. For the PHRF-SCL subscales, the means(±SD) were as follows: Anxiety/Uncertainty: 8.4±1.3 vs. 8.0±1.4, Fatigue/Physical Responses: 7.8±0.8 vs. 7.6±2.1, Autonomic Symptoms: 5.4±1.4 vs. 7.3±1.6, and Depressive Mood/Inadequacy: 5.2±2.3 vs. 5.4±1.4. Among these subscales, Autonomic Symptoms was the only one to exhibit a statistically significant difference (p<0.05).

Table 2 and Table 3 show the results of the subscales for Groups A and B, respectively, as well as the results of independent t-tests and corresponding effect sizes. Only Ruminative Exploration (p=0.692) and Autonomic Symptoms (p=0.513) did not reach statistical significance. Furthermore, of the effect sizes, Extensive Exploration and Depressive Mood/Sufficiency showed the largest differences between the two groups.

**Table 2:** Post-follow-up DIDS-J scores, effect sizes, and mean differences *: independent samples t-test Mean±standard deviation d: effect size; DIDS-J: Dimensions of Identity Development Scale Japanese version

Subscale		Group A (n=4)	Group B (n=4)	d	P-values*
Commitment Formation		16.5±0.6	14.0±0.8	1.23	0.032
Identification with Commitment		16.5±0.6	14.3±1.0	1.31	0.028
Broad Exploration		18.0±0.8	15.0±0.8	1.54	0.008
Deep Exploration		18.0±1.1	14.5±1.3	1.17	0.041
Ruminative Exploration		15.5±1.2	15.0±2.7	0.25	0.692

**Table 3:** Post-follow-up PHRF-SCL scores, effect sizes, and mean differences *: independent samples t-test Mean±standard deviation d: effect size; PHRF-SCL: Public Health Research Foundation Stress Checklist Short Form

Subscale		Group A (n=4)	Group B (n=4)	d	P-values*
Anxiety/Uncertainty		4.5±0.6	7.8±1.3	1.27	0.021
Fatigue/Physical Responses		5.5±1.3	7.5±0.6	0.72	0.043
Autonomic Symptoms		7.8±1.2	9.0±0.8	0.31	0.513
Depressive Mood/Inadequacy		4.5±1.3	9.3±0.6	1.33	0.018

Table 4 and Table 5 summarize the pre- and post-intervention comparisons for each scale in Groups A and B, respectively. In Group A, only Depressive Mood/Inadequacy did not exhibit a statistically significant difference. In Group B, there were no statistically significant differences in Commitment Formation, Identification with Commitment, Broad Exploration, Deep Exploration, Anxiety/Uncertainty, or Fatigue/Physical Responses.

**Table 4:** Pre-post scale scores and mean differences for Group A *: paired t-test Mean±standard deviation DIDS-J: Dimensions of Identity Development Scale Japanese version; PHRF-SCL: Public Health Research Foundation Stress Checklist Short Form

Subscale		Pre	Post	Mean change (post-pre)	P-values*
DIDS-J					
Commitment Formation		13.1±1.1	16.5±0.6	3.4	0.007
Identification with Commitment		12.4±0.8	16.5±0.6	4.1	0.005
Broad Exploration		16.1±2.1	18.0±0.8	1.9	0.034
Deep Exploration		16.2±1.2	18.0±1.8	1.8	0.041
Ruminative Exploration		17.8±2.1	15.5±1.2	-2.3	0.045
PHRF-SCL					
Anxiety/Uncertainty		8.4±1.3	4.5±0.6	-3.9	0.008
Fatigue/Physical Responses		7.8±0.8	5.5±1.3	-2.3	0.032
Autonomic Symptoms		5.4±1.4	7.2±1.2	1.8	0.040
Depressive Mood/Inadequacy		5.2±2.3	4.5±1.3	-0.7	0.217

**Table 5:** Pre-post scale scores and mean differences for Group B *: paired t-test Mean±standard deviation DIDS-J: Dimensions of Identity Development Scale Japanese version; PHRF-SCL: Public Health Research Foundation Stress Checklist Short Form

Subscale		Pre	Post	Mean change (post-pre)	P-values*
DIDS-J					
Commitment Formation		13.7±1.6	14.0±0.8	0.3	0.621
Identification with Commitment		12.7±1.1	13.3±1.0	0.6	0.243
Broad Exploration		15.6±1.6	15.0±0.8	-0.6	0.311
Deep Exploration		15.4±0.8	14.5±1.3	-0.9	0.172
Ruminative Exploration		18.3±1.4	15.0±2.7	-3.3	0.009
PHRF-SCL					
Anxiety/Uncertainty		8.0±1.4	7.8±1.3	-0.2	0.764
Fatigue/Physical Responses		7.6±2.1	7.5±0.6	-0.1	0.845
Autonomic Symptoms		7.3±1.6	9.2±0.8	1.9	0.028
Depressive Mood/Inadequacy		5.4±1.4	9.3±0.4	3.9	0.005

To capture longitudinal changes in both groups, Table 6 shows the changes in scale scores from the end of the preliminary period (one week of system use) to after the washout period and then to approximately nine months later. With respect to DIDS-J subscales, in both groups, Commitment Formation and Identification with Commitment decreased from the preliminary period to the washout period; however, scores increased following system use. For Broad Exploration and Deep Exploration, Group A exhibited increased scores from the preliminary period to the washout period and showed further increases following system use, whereas Group B showed decreases over all time intervals. In Ruminative Exploration, both groups demonstrated increases from the preliminary period to the washout period, followed by a tendency toward lower scores at the end of the study.

**Table 6:** Longitudinal changes in scale scores for Groups A and B Mean±standard deviation DIDS-J: Dimensions of Identity Development Scale Japanese version; PHRF-SCL: Public Health Research Foundation Stress Checklist Short Form

Subscale		Group A (n=4)			Group B (n=4)		
		After the preliminary survey	Pre	Post	After the preliminary survey	Pre	Post
DIDS-J							
Commitment Formation		14.6±0.7	13.1±1.1	16.5±0.6	14.2±1.2	13.7±1.6	14.0±0.8
Identification with Commitment		14.2±1.4	12.4±0.8	16.5±0.6	13.9±0.9	12.7±1.1	13.3±1.0
Broad Exploration		15.4±1.1	16.1±2.1	18.0±0.8	15.7±2.0	15.6±1.6	15.0±0.8
Deep Exploration		14.8±2.3	16.2±1.2	18.0±1.1	15.9±1.6	15.4±0.8	14.5±1.3
Ruminative Exploration		17.6±0.4	17.8±2.1	15.5±1.2	16.2±1.2	18.3±1.4	15.0±2.7
PHRF-SCL							
Anxiety/Uncertainty		6.2±1.2	8.4±1.3	4.5±0.6	6.8±0.6	8.0±1.4	7.8±1.3
Fatigue/Physical Responses		6.9±0.8	7.8±0.8	5.5±1.3	6.4±1.2	7.6±2.1	7.5±0.6
Autonomic Symptoms		4.8±0.4	5.4±1.4	7.2±1.2	4.2±1.4	7.3±1.6	9.2±0.8
Depressive Mood/Inadequacy		4.1±1.6	5.2±2.3	4.5±1.3	3.6±0.9	5.4±1.4	9.3±0.4

Conversely, for PHRF-SCL subscales, all scores increased from the preliminary period to the washout period. At the conclusion of the follow-up period, scores in Group A increased only for Autonomic Symptoms, whereas Group B showed increases in Autonomic Symptoms as well as Depressive Mood/Inadequacy.

## Discussion

This study conducted a nine-month follow-up investigation using the line chart method via a chatbot system to examine how reflective practices might affect identity development and stress, as measured by the DIDS-J and PHRF-SCL.

The intergroup comparisons (Group A vs. Group B) in Table 2 and Table 3 revealed significant differences in the DIDS-J subscales of Commitment Formation, Identification with Commitment, Broad Exploration, and Deep Exploration among participants who continued system use. In the PHRF-SCL, significant differences emerged in Anxiety/Uncertainty, Fatigue/Physical Responses, and Depressive Mood/Inadequacy.

Additionally, the pre-post comparisons within each group (Group A_pre vs. Group A_post and Group B_pre vs. Group B_post) presented in Table 4 and Table 5 indicate that Group A exhibited statistically significant improvement across all DIDS-J subscales　Commitment Formation, Identification with Commitment, Broad Exploration, Deep Exploration, and Ruminative Exploration. In the PHRF-SCL, Anxiety/Uncertainty and Fatigue/Physical Responses improved significantly, whereas Autonomic Symptoms significantly worsened. Although the difference was not statistically significant, the mean change in Depressive Mood/Inadequacy was negative, suggesting a slight decrease in scores. In Group B, Ruminative Exploration on the DIDS-J improved significantly, while Autonomic Symptoms and Depressive Mood/Inadequacy on the PHRF-SCL showed significant deterioration.

These findings suggest that continued use of the system may help maintain good scores on the identity-related subscales of the DIDS-J. Previous reports on chatbot use have raised concerns about the potential for personalized chatbots to reinforce certain biases [16], highlighting the need for research on how chatbots affect user identity. The results of this study provide meaningful insight into this question.

Regarding Autonomic Symptoms, both groups showed comparable degrees of deterioration, which may suggest a broader population-wide trend or the occurrence of specific events affecting the study population as a whole. Additionally, a small sample size and the fact that there was already a significant intergroup difference in Autonomic Symptoms at baseline (Table 1) raise the possibility of selection bias. Previous research also suggests that somatizing tendencies can inflate Autonomic Symptoms scores [15], so it remains possible that this study's participants were prone to such tendencies. Consequently, this investigation cannot conclusively determine the effect of the system on Autonomic Symptoms, warranting further research with an expanded participant pool and broader occupational representation.

In summary, the present study explored the extent to which system use might influence individual identity formation and reduce psychological stress. With respect to identity-related items such as Commitment Formation, Broad Exploration, and Deep Exploration on the DIDS-J, continued system use was associated with significant improvements, suggesting that the system may foster identity formation. In particular, Group A demonstrated improvements in all these domains, raising the possibility that the system exerts a robust impact on identity formation [17].

Moreover, in the PHRF-SCL, marked reductions in Anxiety/Uncertainty and Fatigue/Physical Responses were observed in Group A, implying that system use may mitigate psychological burdens.

On the other hand, both groups exhibited worsening scores on Autonomic Symptoms. This deterioration could be related to the demands of system use itself, occupational factors common to the overall study population, or both [18]. Additionally, selection bias and the occurrence of unusual events affecting participants may have influenced outcomes, particularly given the small sample size and the pre-existing group differences in Autonomic Symptoms. Thus, caution should be exercised in drawing direct conclusions about system effectiveness based on Autonomic Symptoms alone. Future research will require an expanded sample size and a more diverse participant pool.

According to the results presented in Table 6, both groups showed some score fluctuations after the washout period, although certain subscales exhibited no change. These findings indicate that the washout period may not have been optimally set or that external factors (e.g., environmental or personal circumstances) could have intervened during this interval. Moreover, additional long-term investigation is necessary to assess the sustainability of the system's effects. Therefore, future studies should include extended follow-up periods and carefully consider both the long-term efficacy and security aspects of the system [19,20].

Strengths and limitations

Based on the above, this study has several strengths that are worth mentioning. First, the longitudinal study design, spanning approximately nine months, allowed us to assess the long-term effects of chatbot-mediated reflective practice on professional identity formation and stress reduction among healthcare professionals. Second, the use of standardized and validated instruments (DIDS-J and PHRF-SCL) enhanced the reliability and comparability of the findings. The reliability and comparability of the survey results were enhanced. In addition, the randomized parallel-group approach contributes to methodological rigor and reduces potential selection bias.

However, several limitations should also be acknowledged. The first limitation is the small sample size (n=8), which limits the generalizability and statistical power of the results. In addition, participants were limited to nurses and physical therapists from a single facility in Hokkaido, Japan, raising concerns about external validity and applicability to a broader population. Another limitation relates to potential confounding by external factors, such as occupational stressors and personal circumstances that occurred during the long-term study period, which were not fully controlled or measured. Finally, the reliance on self-report measures may introduce biases related to social desirability and accuracy of recall.

Future research should address these limitations by incorporating larger and more diverse participant samples, utilizing additional objective physiological measures, and systematically accounting for external influences to provide a more comprehensive efficacy evaluation of digital mental health interventions.

## Conclusions

This study examined, over approximately nine months, the impact of long-term chatbot system use on professional identity formation and stress reduction among healthcare professionals. The results indicate statistically significant improvements in Commitment Formation and its deepening processes (Broad Exploration, Deep Exploration, and Identification with Commitment) on the DIDS-J, suggesting that reflective practice using the line chart method could promote the development of professional identity. Furthermore, the PHRF-SCL findings showed significant reductions in Anxiety/Uncertainty, Fatigue/Physical Responses, and Depressive Mood/Inadequacy, indicating that the chatbot system may help alleviate psychological burdens. However, no significant intergroup differences emerged in Autonomic Symptoms, implying that population characteristics or external environmental factors may have influenced the results.

Overall, while the present findings suggest that a chatbot system can serve not only as a short-term intervention but also as a long-term psychological support tool, the efficacy appears limited for certain subscales. Future research should expand the study population to include a broader range of occupations beyond healthcare professionals. Moreover, a comprehensive research design that accounts for life events and external factors is warranted. This approach will enable further exploration of digital tools for supporting identity formation and guide the incorporation of multicultural and multilingual elements in subsequent system development.

## Appendices

**Table 7:** The Japanese version of the Dimensions of Identity Development Scale Please select the response that best describes your situation: 1 = Does not apply; 2 = Does not apply much; 3 = Neutral; 4 = Somewhat applies; 5 = Applies

Factor	Question	1	2	3	4	5
Commitment Making	I have decided what kind of life I want to lead.					
	I have chosen and decided what to do with my life.					
	I have a plan for what I will continue doing.					
	I have a goal for the life I want to pursue.					
	I can make a firm decision to create a life that satisfies me.					
Identification with Commitment	My future plans align with the direction I want my life to take and what I consider important.					
	I feel that my future plans are the right ones for me.					
	If I have future plans, I feel they are valuable.					
	The goals of my path and career are satisfying to me.					
	The life path I want to follow truly suits me.					
Exploration in Breadth	I actively think about what kind of life I want to pursue.					
	I consider various possibilities regarding what I will continue doing.					
	I think about various directions for my career and way of life.					
	I broadly explore career and life paths that seem good for me.					
	I consider many different paths that may suit me.					
Exploration in Depth	I think about whether my future career and life goals truly suit me.					
	I try to understand how others perceive the life I want to pursue.					
	I reflect on the future plans I have decided to follow.					
	I think about whether my future plans truly align with what I want.					
	I discuss my chosen path and plans with others.					
Ruminative Exploration	I am unclear about what I really want to do in life.					
	I feel lost about what kind of life I want to pursue.					
	I find it difficult to choose my career path.					
	I constantly reconsider what kind of life I should lead.					
	I keep doubting the life path I want to follow.					
